# Bone graft absorption complication following cranioplasty: A retrospective institutional study

**DOI:** 10.3892/mi.2024.156

**Published:** 2024-04-16

**Authors:** Charalampos Gatos, George Fotakopoulos, Vasiliki Epameinondas Georgakopoulou, Theodosis Spiliotopoulos, Pagona Sklapani, Nikolaos Trakas, Adamantios Kalogeras, Kostas N. Fountas

**Affiliations:** 1 Department of Neurosurgery, General University Hospital of Larissa, 41221 Larissa, Greece; 2 Department of Pathophysiology, National and Kapodistrian University of Athens, 11527 Athens, Greece; 3 Department of Biochemistry, Sismanogleio Hospital, 15126 Athens, Greece

**Keywords:** cranioplasty, decompressive craniectomy, traumatic brain injury, cranial reconstruction

## Abstract

The aim of the present retrospective study was to confer the factors that are related to bone graft absorption and affect the outcomes of patients following cranioplasty (CPL). The present retrospective study includes cases of patients that underwent CPL between February, 2013 and December, 2022. All participants had a follow-up period of 1 to 10 years from the day of discharge from the hospital. In total, 116 (62.3%) of the 186 patients that underwent decompressive craniectomy (DC) were enrolled in the present study for CPL. A total of 109 (93.9%) patients were included in group A, and 7 (6.0%) patients were included in group B. On the whole, the results of the present study suggest that a CPL after 2.5-7.7 months of DC increases the possibility of bone absorption.

## Introduction

Decompressive craniectomy (DC) is a surgical procedure which as long been used with varying usefulness for the treatment of refractory intracranial hypertension for a wide range of pathologies (1-5). Although the complications associated with this technique and the functional outcomes of surviving patients have not yet been fully determined (6,7), DC can be a lifesaving technique in the presence of medically intractable elevations of intracranial pressure, and may consequently increase the length of stay in intensive care units (8).

However, the prolonged exposure of skull defects has been associated with various neurological manifestations, including the immediate effects of atmospheric pressure on the soft brain tissue, obstructions and hydrodynamic changes in cerebrospinal fluid, and modifications in cerebral blood flow and metabolism (6,7,9,10).

Cranioplasty (CPL) is a procedure used for reconstructing skull deficits, providing cerebral protection, and enhancing the cosmetic effect (11). In addition, CPL may aid in the neurological recovery of patients due to its physiological effects on the cranial vault, allowing for a more effective rehabilitation process (11). Nevertheless, critical clinical questions remain, including significant post-operative morbidity, various complications in neurological recovery and outcomes, infections, seizures, hematomas, the influence timing has on these factors, the selection of materials, overall cost-effectiveness and bone graft absorption (BGA) (12,13).

Concerning the type of bone graft, above all, the advantage of autologous as opposed to heterologous bone grafts is that there is no rejection (14). On the other hand, BGA is a severe complication (15). In particular, the skull bone has a higher tendency for absorption compared with other parts of the body. If implanted, skull graft resorption continues, and the bone graft may break down, necessitating further surgery (15). In the literature, there are several issues on whether early CPL, the age of the patient, or the type of bone graft could lead to resorption (15,16).

The aim of the present retrospective study was to confer the factors that are related to BGA and may affect the outcomes of patients following CPL.

## Patients and methods

### Study design and population

The present study constitutes a single-center, retrospective study of patients who underwent CPL. The population of interest was defined as all patients that underwent CPL at a local institution (University Hospital of Larissa, Larissa, Greece) between February, 2013 and December, 2022. The Institutional Review Board (IRB) of the University of Thessaly, Greece, and the University Hospital of Larissa approved the study (IRB no. 2542/21-01-2021, finalized by the 28th General Assembly on January 28, 2021). Written informed consent was obtained from all included patients or their next-of-kin before surgery, and for under-age patients, consent was obtained from their parents or legal guardians.

**Figure 1:**
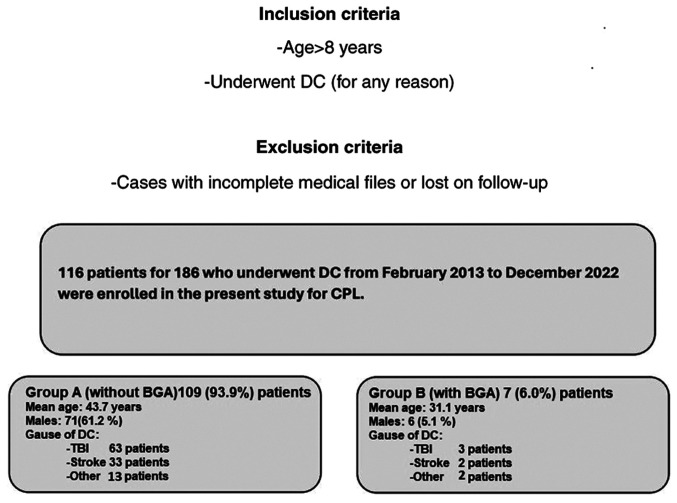
Flow chart of the study participants. DC, decompressive craniectomy; CPL, cranioplasty; BGA, bone graft absorption.

In total, of the 186 patients that underwent DC, 116 patients proceeded to the University Hospital of Larissa for CPL, and 7 (6.0%) patients developed BGA during the follow-up. In the final pool, 116 patients were included, and these patients were divided into two groups. Data collection was performed, and the data were reviewed and analyzed by two physicians (GF and CG) on the basis of the following inclusion criteria: Patients aged >8 years old who underwent DC (for any reason) and subsequent CPL between 2013 and 2022. Cases with incomplete medical files and cases lost to follow-up were excluded (Fig. 1).

### Clinical data

**Figure 2:**
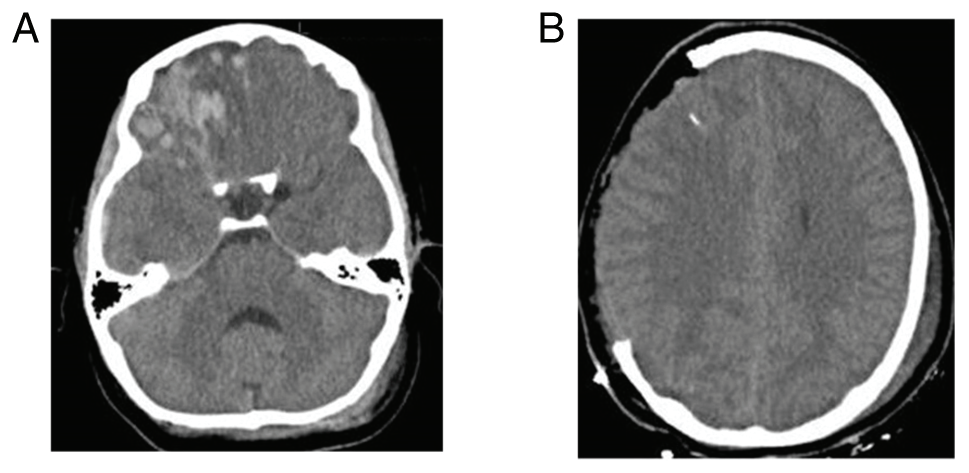
A 9-year-old female patient was admitted with a Glasgow Coma Scale score of 10 following severe traumatic brain injury and anisocoria (pupil right>left). (A) The first (pre-operative) computed tomography scan revealed the following: Brain contusions, edema and subdural hematoma, with an elevated intracranial pressure (>22 mmHg). (B) Post-operative computed tomography scan following decompressive craniectomy.

**Figure 3:**
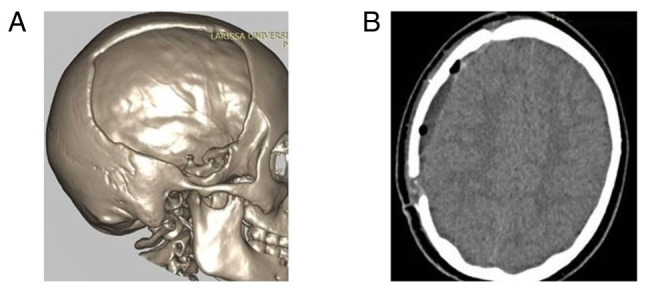
Images of the same patient as depicted in Fig. 2 are shown. (A) Post-operative 3D volume rendering following decompressive craniectomy. (B) Post-operative computed tomography scan following cranioplasty performed 9 months later with the cryopreservation of the autologous bone graft at -80˚C.

**Figure 4:**
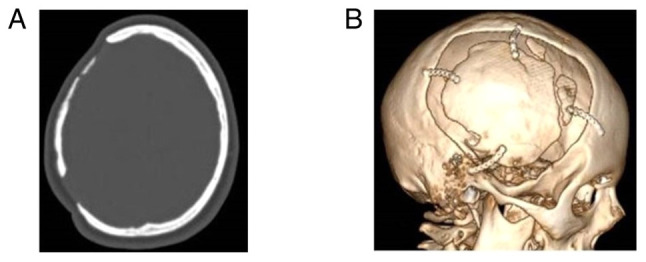
Images of the same patient as depicted in Figs. 2 and 3 are shown. (A) Computed tomography scan (bone) and (B) 3D volume rendering during follow-up demonstrating bone graft absorption, cosmetic disfigurement; lesion, >1 cm and remnant thickness of bone flap <50% of the contralateral skull region.

The patients were divided into two groups, namely group A, which included patients treated with CPL who did not develop BGA during the follow-up period, and group B, which included those who developed BGA. These groups were identified based on the following demographic, clinical and radiographic data that were retrieved from the medical archives when available: Age, sex, cause of DC [traumatic brain injury (TBI), stroke, other neurosurgical entities that required DC, such as subarachnoid hemorrhage, tumor, brain abscess, cerebral venous sinus thrombosis and patients developed intracerebral hemorrhage], Glasgow Coma Scale (GCS) and Karnofsky Performance Scale (KPS) of admission, history of diabetes and hypertension, site of CPL [one site fronto-temporo-parietal (FTP), bilateral FTP, bilateral frontal], time from DC to CPL, type of bone graft (heterologous or autologous), grafts with fractures or fragments, and peri-operative complications such as infections and hematomas (Table I). All participants had a follow-up period of 1 to 10 years from the day of discharge from the hospital. Patient outcomes were evaluated using a computer tomography (CT) scan and a complete neurological examination at 6 months, 1 year, and 3 or 6 years following discharge from the hospital. The primary outcome was defined as neurological deterioration, and the secondary outcomes were hospital stay and mortality. The CPL implant material was heterologous or autologous and cryopreserved at -83˚C and taken out to thaw at room temperature 2 h before the intervention. Images of a case that was evaluated are presented in Fig. 2, Fig. 3 and Fig. 4.

### Statistical analysis

Statistical analyses were performed using the Statistical Package for the Social Sciences (SPSS 11; SPSS, Inc.). The normality of the distribution of variables was assessed using the Shapiro-Wilk test. Categorical variables were compared between groups using the Fisher's exact test, and continuous data were compared using the Mann-Whitney U test. Receiver operating characteristic (ROC) analysis was used to reveal the factors that are related to BGA and affect the outcomes of patients following CPL. A P-value <0.05 was considered to indicate a statistically significant difference.

## Results

In total, 116 (62.3%) of the 186 patients that underwent DC were enrolled in the present study for CPL. A total of 109 (93.9%) patients were included in group A, and 7 (6.0%) patients were included in group B. Of the 116 patients included, 77 (66.3%) were males, and the median age was 42.5 years. The baseline characteristics of the study participants are presented in Table I. The outcomes of the patients are presented in Table II.

Univariate analysis revealed that there was a statistically significant difference in the time from DC to CPL, infections and hematoma as peri-operative complications between the participants who developed BGA and those who did not develop BGA (P<0.05, Table III).

**Figure 5:**
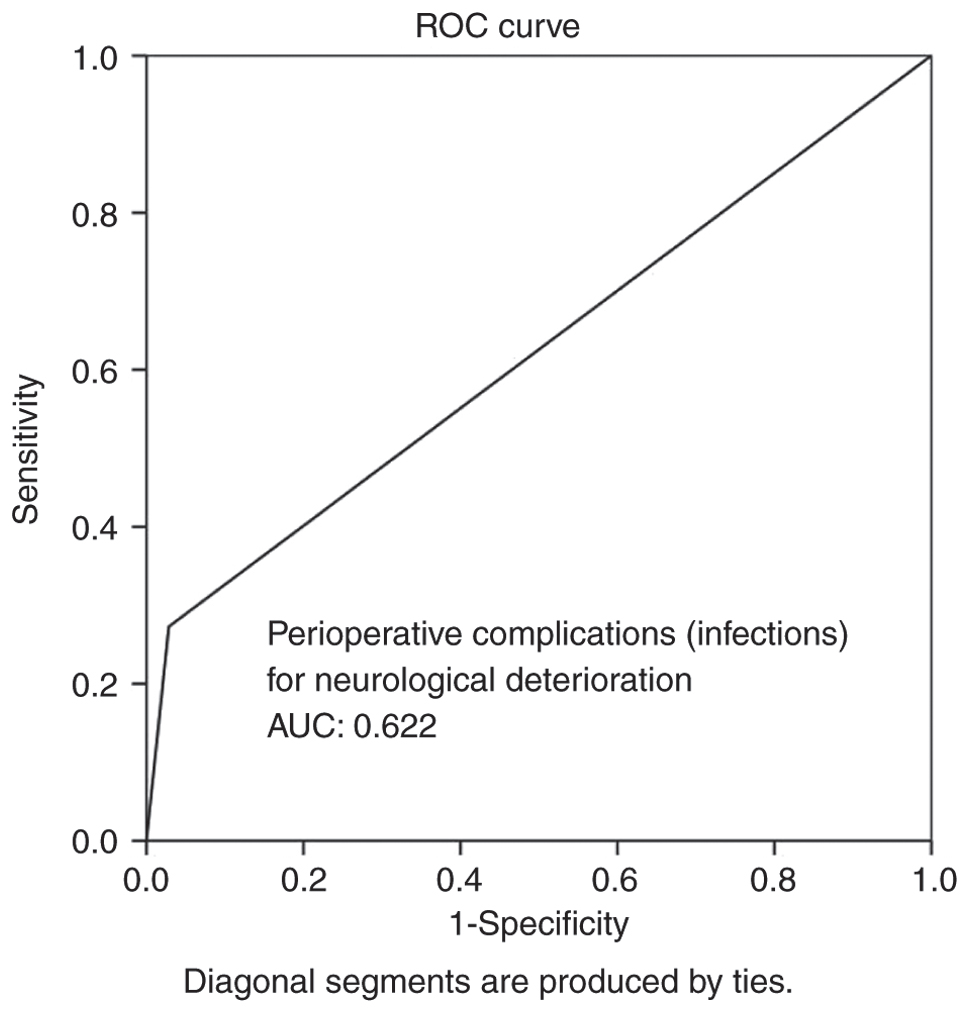
ROC curve for peri-operative complications (infections), predicting bone graft absorption during follow-up. AUC, 0.622. AUC, area under the curve; ROC, receiver operative characteristic.

**Figure 6:**
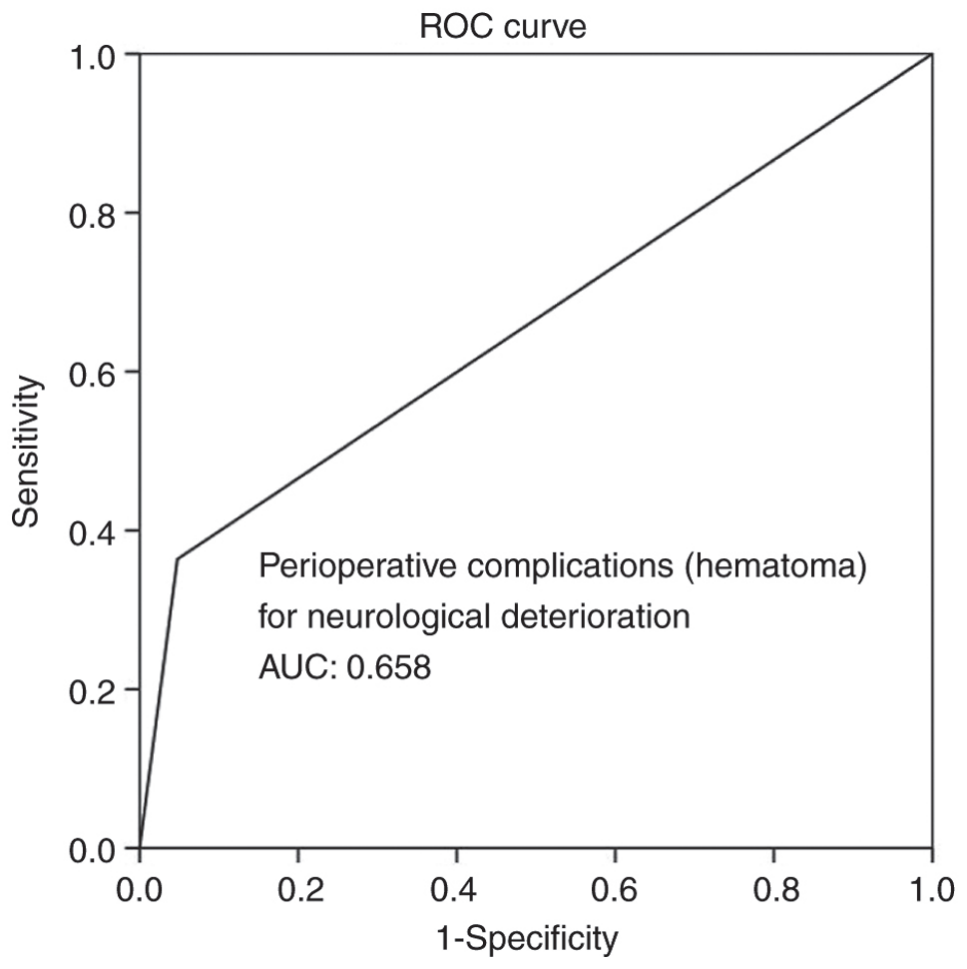
ROC curve for peri-operative complications (hematoma), predicting bone graft absorption during follow-up. AUC, 0.658. AUC, area under the curve; ROC, receiver operative characteristic.

**Figure 7:**
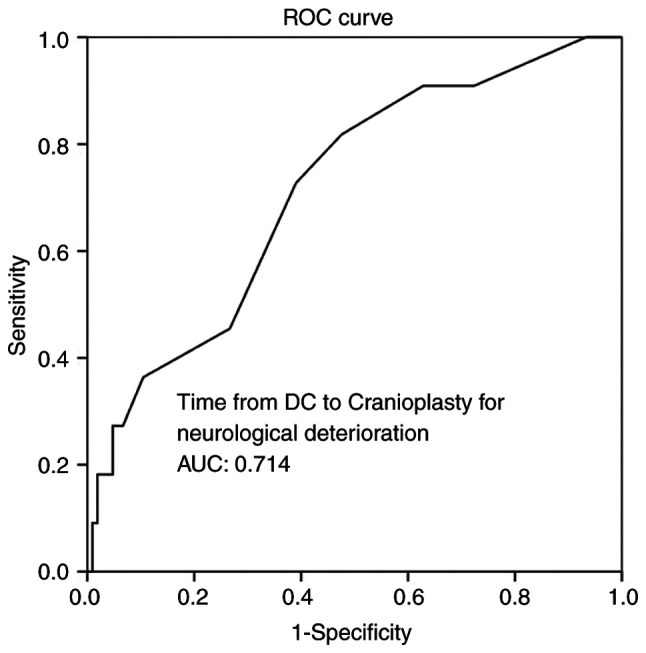
ROC curve for time from DC to cranioplasty, predicting bone graft absorption during follow-up. AUC, 0.714. AUC, area under the curve; ROC, receiver operative characteristic; DC, decompressive craniectomy.

Multivariate analysis (Table IV) revealed that time from DC to CPL, infections and hematoma as peri-operative complications were all independent factors associated with BGA during follow-up (P<0.05 for all three parameters). Overall, ROC analysis demonstrated that infections and hematoma as peri-operative complications exhibited the optimal performance to predict BGA, as evaluated by an area under the curve standard error [AUC (SE)] of [0.622 (0.10) and (P=0.184)] and [0.658 (0.10) and (P=0.085)], respectively (Table V, and Figs. 5 and 6). In addition, ROC analysis demonstrated that, among the variables, a time from DC to CPL of 2.5 months with 100% sensitivity and 93.3% specificity exhibited a better dispersion to predict BGA, as evaluated by an area under the curve standard error [AUC (SE)] of [0.714 (0.79)] and (P=0.020) (Table V and Fig. 7).

## Discussion

The results of the present study suggest that a CPL after 2.5-7.7 months of DC increases the possibility of bone absorption. Additionally, the presence of post-operative infections and hematoma, not alone but in combination with the time from DC to CPL factor, was shown to contribute decisively to the absorption of the bone graft.

### Bone graft material

The type of bone graft used for CPL can be heterogeneous or autologous, and the material can be variable, as there are no indications as to the ideal material which should be used for CPL (15). Other than the autologous bone, metal plates, hydroxyapatite (HA), poly(methyl methacrylate, HA cement and polyethylene have been implanted in order to perform such necessities (17). The present study did not reveal any statistically significant differences among the types or materials that were used for CPL.

### Complications: infections and hematoma

The rate complications associated with CPL has a wide range of differences among several studies in the literature. The infection rate has been reported to be 6 to 12%, which in numerous cases leads to implant removal and, together with hematomas, is the most frequently reported (18-22). The findings of the present study demonstrated that the rates of infection and hematoma were 6 and 9%, respectively, and not alone, but in combination with the time from DC to CPL, were shown to contribute decisively to the development of BGA.

### Time from DC to CPL

As regards CPL, the time of the bone graft re-implantation is one of the most commonly debated issues. There are studies reporting that early bone graft implantation is related to various complications and a poorer outcome (22,23). Along with the complications in the early stages of CPL, hydrocephalus was the most common due to its association with other factors, such as size and the cause of DC. In addition, infections constitute another severe post-CPL complication, mainly if it is performed before 60 days have passed after DC (22). On the other hand, CPL performed at a late stage is associated with the same complications, and there are no indications as to the optimal time frame for performing CPL following DC (24,25).

However, some studies have mentioned that 3-6 months is suitable for bone graft preservation (24,25). In the present study, the time from DC to CPL was an independent parameter predicting BGA, and restoration after 2.5-7.7 months increases the possibility of bone absorption. Thus, the results presented herein suggest that in clinical practice, 2.5-7.7 months constitute the most suitable time interval for performing CPL following DC without the various complications related to early bone graft implantation, such as infections, as well as with a minimal risk of BGA, which is usually related to CPL performed at a late stage.

### Patient's age

Apart from the time interval between DC and CPL, the age of the patients represents another parameter in the development of BGA (24). Thus, in pediatric research, BGA has been found at a high rate, reaching 50% of patients with CPL at a mean follow-up of 4.8 months (26). The independent risk factors for BGA accountably included skull fracture, underlying contusion, post-traumatic hydrocephalus, and an age of 2.5 years (26). The present study demonstrated that even the young age of the patients (<19 years) was not a factor in predicting BGA during the follow-up period following CPL.

The present study had several limitations that should be mentioned. The main limitation was that it was performed in a single center, and its retrospective nature was related to possible errors in collecting and interpreting the data from the clinical history. Another limitation also was the small sample size in group B (n=7), and thus the power to detect significant differences is questionable. In addition, the neurological outcome of patients following DC and subsequent CPL depends on the underlying initial pathology.

In conclusion, although CPL is a relatively straightforward type of surgery from a technical standpoint, it is not come without controversies. The results of the present study suggest that CPL performed after 2.5-7.7 months of DC increases the possibility of bone absorption. Additionally, the presence of post-operative infections and hematoma, not alone, but in combination with the time from DC to CPL factor, was shown to contribute decisively to the absorption of the bone graft. This sequence provides a strong justification for further extensive prospective clinical investigations into the prevention of BGA following CPL.

## Figures and Tables

**Table I:** Baseline demographic characteristics of the patients.

Parameters	All patients, n=116 (100%)	Group A, n=109 (93.9%)	Group B, n=7 (6.0%)	P-value
Age, mean ± SD (years)	42.5±14	43.7±14	31.1±8.7	**0.024**
Sex (male), n (%)	77 (66.3)	71 (61.2)	6 (5.1)	0.264
Cause of DC				
TBI, n (%)	66 (56.8)	63 (54.3)	3 (2.5)	0.439
Stroke, n (%)	35 (30.1)	33 (28.4)	2 (1.7)	0.924
Other^a^, n (%)	15 (12.9)	13 (11.2)	2 (1.7)	0.203
GCS score of admission, mean ± SD	10.0±2.3	9.1±2.1	77.8±6.3	0.310
KPS score of admission, mean ± SD	75.9±4.6	19.9±7	18.4±6	0.495
Diabetes mellitus, n (%)	9 (7.7)	9 (7.7)	0 (0)	0.429
Hypertension, n (%)	17 (14.6)	17 (14.6)	0 (0)	0.258
Site of cranioplasty				
One-site FTP, n (%)	99 (85.3)	94 (81.0)	5 (4.3)	0.283
Bilateral frontal, n (%)	7 (6.0)	7 (6.0)	0 (0)	0.489
Bilateral FTP, n (%)	10 (8.6)	8 (6.8)	2 (1.7)	0.052
Time from DC to cranioplasty, mean ± SD (months)	6.31±3.9	6.13±3.8	9.14±4.9	**0.034**
Type of graft				
Autologous, n (%)	84 (72.4)	79 (68.1)	5 (4.3)	0.952
Heterologous, n (%)	32 (27.5)	30 (25.8)	2 (1.7)	0.952
Grafts with fragments or fractures, n (%)	9 (7.7)	9 (7.7)	0 (0)	0.429
Peri-operative complications				
Infections, n (%)	6 (5.1)	6 (5.1)	0 (0)	0.524
Hematoma, n (%	9 (7.7)	6 (5.1)	3 (2.5)	**<0.05**

Values in bold font indicate statistically significant differences (P<0.05).

^a^Other, refers to neurosurgical entities that required DC, such as subarachnoid hemorrhage, tumor, brain abscess, cerebral venous sinus thrombosis event and patients developed intracerebral hemorrhage. KPS, Karnofsky Performance Scale; TBI, traumatic brain injury; GCS, Glasgow Coma Scale; SD, standard deviation; DC, decompressive craniectomy; FTP, fronto-temporoparietal.

**Table II:** Outcomes of patients following cranioplasty.

Parameters	All patients, n=116 (100%)	Group A n=109 (93.9%)	Group B n=7 (6.0%)	P-value
Mortality, n (%)	5 (4.3)	5 (4.3)	0 (0)	0.562
Neurological deterioration, n (%)	11 (9.4)	6 (5.1)	5 (4.3)	**<0.05**
Duration of hospital stay, mean ± SD (days)	5.9±0.9	5.8±0.9	6.4±0.9	0.161

Values in bold font indicate statistically significant differences (P<0.05). SD, standard error.

**Table III:** Univariate analysis for neurological deterioration.

Parameters	No neurological deterioration, n=105 (90.5%)	With neurological deterioration, n=11 (9.4%)	P-value
Age, mean ± SD (years)	43.1±14	41.0±13	0.591
Sex (male), n (%)	69 (59.4)	8 (6.8)	0.639
Cause of DC			
TBI, n (%)	62 (53.4)	4 (3.4)	0.148
Stroke, n (%)	29(25)	6 (5.1)	0.064
Other, n (%)	14 (12.0)	1 (0.8)	0.690
GCS score of admission, mean ± SD	10.1±2.3	9.6±1.9	0.659
KPS score of admission, mean ± SD	75.8±4.5	75.9±4.6	0.442
Diabetes mellitus, n (%)	9 (7.7)	0 (0)	0.312
Hypertension, n (%)	17 (14.6)	0 (0)	0.149
Site of cranioplasty			
One-site FTP, n (%)	90 (77.5)	9 (7.7)	0.728
Bilateral frontal, n (%)	7 (6.0)	0 (0)	0.377
Bilateral FTP, n (%)	8 (6.8)	2 (1.7)	0.235
Time from DC to cranioplasty, mean ± SD (months)	5.9±3.6	9.4±5.8	**0.019**
Type of graft			
Autologous, n (%)	75 (64.6)	9 (7.7)	0.463
Heterologous, n (%)	28 (24.1)	4 (3.4)	0.494
Grafts with fragments or fractures	9 (7.7)	0 (0)	0.312
Peri-operative complications			
Infections, n (%)	3 (2.5)	3 (2.5)	**0.001**
Hematoma, n (%)	7 (6.0)	2 (1.7)	**0.034**
Duration of hospital stay, mean ± SD (days)	5.8±0.9	6.0±1.0	0.564

Values in bold font indicate statistically significant differences (P<0.05). KPS, Karnofsky Performance Scale; TBI, traumatic brain injury; GCS, Glasgow Coma Scale; SD, standard deviation; DC, decompressive craniectomy; FTP, fronto-temporoparietal.

**Table IV:** Multivariate analysis for neurological deterioration.

			95% CI for Exp(B)	
Parameter	P-value	Exp(B)	Lower	Upper
Time from DC to cranioplasty, mean ± SD (months)	**0.003**	0.245	0.006	0.030
Peri-operative complications				
Infections	**<0.05**	0.359	0.266	0.682
Hematoma	**<0.05**	0.350	0.211	0.556

Values in bold font indicate statistically significant differences (P<0.05). SD, standard deviation; CI, conﬁdence interval; DC, decompressive craniectomy.

**Table V:** ROC analysis for neurological deterioration.

Parameters	Area	Std. error	95% CI lower-upper	P-value
Time from DC to cranioplasty, mean ± SD (months)	0.714	0.079	0.560-0.868	**0.020**
Peri-operative complications				
Infections	0.622	0.101	0.424-0.821	0.184
Hematoma	0.658	0.101	0.461-0.855	0.085

Values in bold font indicate statistically significant differences (P<0.05). SD, standard deviation; CI, conﬁdence interval; DC, decompressive craniectomy.

## Data Availability

The datasets used and/or analyzed during the current study are available from the corresponding author on reasonable request.
